# Six promising drug repurposing candidates for Alzheimer’s disease and their sex-specific mechanisms and efficacy

**DOI:** 10.4103/NRR.NRR-D-25-00256

**Published:** 2025-08-13

**Authors:** Maria E. Figueiredo-Pereira, Peter A. Serrano, Patricia Rockwell

**Affiliations:** 1 Department of Biological Sciences, Hunter College CUNY, New York, NY, USA; 2 Department of Psychology, Hunter College CUNY, New York, NY, USA

**Keywords:** Alzheimer’s disease, BT-11, diazoxide, dibenzoylmethane, drug repurposing, high-throughput drug approach, ibudilast, RG2833, TgF344-AD rat model, timapiprant

## Abstract

Alzheimer’s disease is a neurodegenerative disorder that leads to progressive memory loss, cognitive decline, and behavioral changes. Despite ongoing research, its exact causes and effective treatments remain elusive. Traditional approaches have focused on symptom management, but breakthroughs in bioinformatics and high-throughput drug screening are offering new pathways to potential therapies. This review highlights our recent efforts to identify novel drug candidates for Alzheimer’s disease by leveraging computational methods and large-scale biological datasets. Our work introduces two key innovations in Alzheimer’s disease research: addressing sex-specific differences and leveraging drug repurposing for accelerated treatment discovery. By combining sex-stratified preclinical data with machine learning and *in vivo* validation, we improve translational relevance and support precision medicine. Using the TgF344-AD rat model, which mimics human Alzheimer’s disease spatial memory deficits and pathology, we explored the efficacy of various US Food and Drug Administration–approved and investigational drugs. These included ibudilast, timapiprant, RG2833, diazoxide/dibenzoylmethane (combined), and BT-11, which targeted key Alzheimer’s disease–related molecular pathways such as amyloid-beta plaques, Tau tangles, and neuroinflammation. These drugs, at various stages of development, offer hope for not only managing symptoms but also addressing the underlying mechanisms of Alzheimer’s disease. This review underscores the need for a multifaceted approach to Alzheimer’s disease treatment, combining symptom relief with disease modification.

## Introduction

Alzheimer’s disease (AD) is a complex neurodegenerative disorder that affects millions worldwide, leading to progressive memory loss, cognitive decline, and behavioral changes. The precise causes and underlying mechanisms of AD remain largely unclear, and current treatments offer only limited symptom relief without significantly altering disease progression (Madar et al., 2024). Traditional therapeutic strategies have focused on broad-spectrum medications, which have had minimal success in modifying the course of the disease (Peng et al., 2023).

Recent advances in bioinformatics and computational biology have opened new pathways for identifying potential AD treatments. High-throughput drug screening techniques are increasingly used to repurpose both US Food and Drug Administration (FDA)–approved and investigational drugs by evaluating their ability to target molecular pathways implicated in AD (Zulhafiz et al., 2025). These approaches integrate large-scale biological datasets, including genomic, transcriptomic, and proteomic data, to identify promising drug candidates and predict their therapeutic effects (Xu et al., 2022). By combining molecular insights with advanced algorithms, researchers aim to accelerate the development of effective, disease-modifying treatments.

In this review, we summarize our research focused on identifying and evaluating novel drug candidates for AD using the TgF344-AD rat model. This model is widely recognized for its ability to replicate key features of human AD, including spatial memory deficits, cognitive impairments, neuronal loss, amyloid plaque accumulation, and Tau pathology (Cohen et al., 2013). Its comprehensive resemblance to human AD pathology makes it an ideal platform for preclinical drug testing, allowing us to rigorously assess both efficacy and safety while evaluating biomarker responses and cognitive outcomes.

An important innovation in our research is the deliberate investigation of gender differences in AD pathology and treatment response, an area that has been underexplored. Emerging evidence suggests that biological sex significantly influences disease onset, progression, and therapeutic outcomes in AD, with women showing higher prevalence and often more severe cognitive decline than men (Mosconi et al., 2021). In our preclinical assessments using the TgF344-AD model, we stratified data by sex to identify sex-specific biomarkers and differential responses to drug candidates. This stratification enhances the translational relevance of our findings and supports the development of personalized interventions.

Additionally, our focus on drug repurposing, the systematic evaluation of FDA-approved and investigational drugs for new therapeutic applications, represents a highly efficient and cost-effective strategy. Drug repurposing leverages existing pharmacokinetic and safety data, thereby accelerating the timeline from discovery to clinical application (Zhan et al., 2022). Our integration of machine learning predictions with *in vivo* testing validates computational outcomes and helps prioritize compounds that may have been overlooked in traditional pipelines. This dual innovation of examining gender-based differences and adopting a strategic repurposing framework aligns with precision medicine goals and addresses key gaps in current AD research.

To identify potential therapeutic compounds, we employed a machine learning-driven approach, leveraging computational tools to analyze extensive datasets and predict drug-target interactions (Lim et al., 2016; Wang et al., 2018; Wu et al., 2022). This enabled us to identify compounds with high potential to engage key pathological targets such as amyloid-beta aggregates, Tau tangles, and neuroinflammatory pathways.

Our focus in this review includes several FDA-approved and investigational drugs that show promise for repurposing in AD treatment:

• *Ibudilast*, a phosphodiesterase inhibitor, exhibits anti-inflammatory and neuroprotective properties, making it a strong candidate for AD intervention (Oliveros et al., 2023).

• *Timapiprant*, a selective prostaglandin D2 receptor antagonist, targets neuroinflammation—a central aspect of AD pathology (Wallace et al., 2022).

• *RG2833*, a histone deacetylase inhibitor, is under investigation for its ability to modulate gene expression and potentially delay cognitive decline (Ndukwe et al., 2025).

• *Diazoxide*, commonly used for hypoglycemia, has been studied for its role in modulating mitochondrial function and stress response pathways relevant to AD (Wallace et al., 2024).

• *Dibenzoylmethane* appears to interfere with protein aggregation and amyloid-beta deposition, addressing two hallmarks of AD pathology (Wallace et al., 2024).

• *BT-11*, a novel investigational compound, has shown early neuroprotective effects in preclinical models (Birnbaum et al., 2024).

These compounds represent a diverse set of mechanisms, offering hope for treatments that move beyond symptom management to address the underlying biology of AD. Collectively, they reflect a growing commitment to multifaceted, data-driven strategies in the fight against AD.

In conclusion, our review highlights the urgent need for innovative and comprehensive therapeutic approaches to AD. By integrating computational modeling, animal research, and molecular biology, we aim to contribute to the development of more effective and lasting treatments that offer real hope for patients and caregivers alike.

## Search Strategy

Studies mentioned in this narrative review were searched on the PubMed database using these keywords: Alzheimer’s disease, drug repurposing, high-throughput drug approach, ibudilast, timapiprant, RG2833, diazoxide, dibenzoylmethane, BT-11, TgF344-AD rat model. All years were included in the search.

## TgF344-Alzheimer’s Disease Rat Model

In these studies, all drugs were evaluated on male and female TgF344-AD rats (Cohen et al., 2013). This rat model expresses mutant human “Swedish” amyloid precursor protein (APPsw) and a Δ exon 9 presenilin-1 (PS1ΔE9) at levels 2.6 and 6.2 times higher than the endogenous rat proteins, respectively (Cohen et al., 2013). The prion promoter drives the expression of these human genes (Cohen et al., 2013). These mutations lead to the accumulation of amyloid plaques, tauopathy, gliosis, neuronal loss, and cognitive deficits in a progressive age-dependent manner, a hallmark of AD pathology that is difficult to reproduce in animal models (Cohen et al., 2013; Saré et al., 2020). The TgF344-AD rat model thus meets the critical need for a next-generation rodent model for both basic and translational research, making it ideal for our studies. TgF344-AD rats were purchased from the Rat Resource and Research Center (RRRC, Columbia, MO, USA) and were housed in pairs on a 12-hour light/dark cycle with *ad libitum* access to food and water. All animal procedures were approved by the Institutional Animal Care and Use Committee (IACUC) at Hunter College.

Drug treatments were administered via *ad libitum* 5001 Purina rodent chow (from Research Diets, Inc., New Brunswick, NJ, USA). Each drug was assessed at a predetermined concentration based on literature, and its effects were compared to a control chow with no drug.

## Cognitive Performance and Neuropathological Features in the TgF344-Alzheimer’s Disease Rat Model

**Figure 1:**
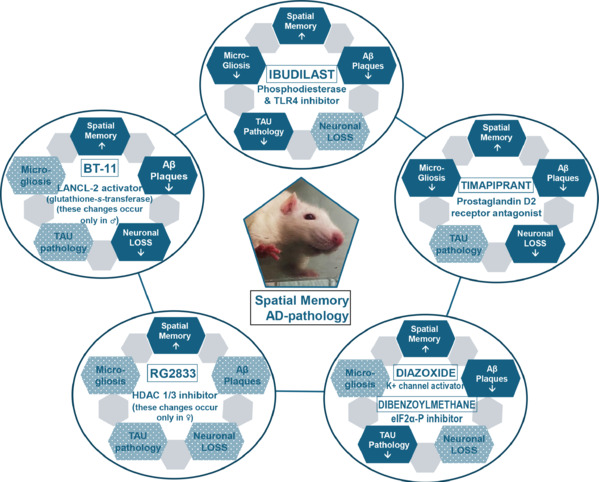
Schematic summary of the effects of five pharmacological treatments on AD-related phenotypes in TgF344-AD rats. Each treatment [ibudilast, timapiprant, DZ/DIB (combination therapy), RG2833, and BT-11] is illustrated within individual circles arranged in a pentagonal layout, with the pharmacological target of each compound indicated at the center of its respective circle. Where applicable, sex-specific treatment effects are noted. A representative image of a TgF344-AD rat is shown at the center of the pentagon, along with two core AD features assessed in this study: spatial memory and AD pathology. Drug-induced changes in spatial memory performance, Aβ plaque burden, neuronal loss, TAU pathology, and microgliosis were evaluated relative to untreated TgF344-AD controls. Directional arrows indicate the nature of drug effects: upward arrows (↑) denote an increase, while downward arrows (↓) signify a decrease in the measured parameter. Dark blue hexagons indicate a beneficial effect on a specific AD pathology, whereas light blue hexagons denote no observable effect. For comprehensive descriptions of the impact of each drug, refer to our corresponding publications cited under references. AD: Alzheimer’s disease; Aβ: amyloid-beta; eIF2α-P: phosphorylated eukaryotic initiation factor 2; HDAC: histone deacetylase; LANCL-2: lanthionine synthetase C-like 2; TLR4: toll-like receptor 4.

Our studies (listed for each drug under references) provide significant insights into the role of sex differences in the cognitive outcomes and neuropathological progression of AD models, particularly in TgF344-AD rats. Our data reveal the complex interactions between treatment, cognition, and brain pathology, highlighting how these relationships differ between males and females, and suggesting that therapeutic strategies may need to be tailored based on sex-specific biological factors. We focus on cognitive performance, neuropathological markers (including amyloid plaques, Tau, neuronal loss, and microgliosis), treatment outcomes, and the underlying biological mechanisms that contribute to the observed sex differences (**Figure 1**).

## Cognitive Performance in TgF344-Alzheimer’s Disease rats

One of the most striking findings across the studies is the difference in cognitive performance between male and female TgF344-AD rats. Despite the presence of significant amyloid plaque load, female TgF344-AD rats consistently outperformed males in the active place avoidance task (Chaudry et al., 2022). This performance difference is particularly noteworthy given that female TgF344-AD rats do not exhibit the same extent of cognitive decline as their male counterparts, even with a comparable amyloid plaque burden. The cognitive resilience observed in females contrasts with the pronounced cognitive deficits in males, suggesting that sex may play a critical role in how AD pathology affects brain function (Chaudry et al., 2022; Ndukwe et al., 2025). These findings align with a growing body of literature suggesting that females may have a form of inherent protection against the cognitive decline typically associated with AD, a phenomenon potentially linked to hormonal factors, including estrogen, which is known to exert neuroprotective effects (Ali et al., 2023; Andy et al., 2024; Suganya et al., 2024). Conversely, males appear to be more susceptible to the pathological effects of amyloid plaques, which could be due to sex-specific differences in brain structure, neuroinflammation, or other factors (Chaudry et al., 2022). This raises the possibility that treatment strategies for AD may need to consider sex as a crucial factor when assessing cognitive outcomes.

## Neuropathological Features: Amyloid Plaques, Mcrogliosis, Hyperphosphorylated Tau

Neuropathological analysis of TgF344-AD rats showed sex-specific differences in AD progression. Males had a greater accumulation of amyloid plaques, but cognitive decline was more closely linked to hippocampal neuronal loss than plaque burden (Birnbaum et al., 2024). This suggests that the accumulation of amyloid-beta (Aβ) plaques alone may not fully explain the cognitive decline seen in AD and that other factors, such as neuronal integrity and synaptic plasticity, play important roles in determining cognitive function.

Microgliosis, especially in the hippocampus, was also more severe in males, correlating with worse cognitive outcomes. In contrast, females showed milder microglial activation and more stable cognition despite similar Aβ loads (Ndukwe et al., 2025). These sex differences in microgliosis could contribute to the disparity in cognitive outcomes between males and females. Anti-inflammatory treatment with ibudilast may help mitigate these effects in AD patients (Oliveros et al., 2023).

Findings on Tau pathology are mixed: some studies report higher p-Tau in females (Niu et al., 2021; Tsiknia et al., 2022; Coughlan et al., 2025), others in males (Joynes et al., 2025), with testosterone loss worsening Tau accumulation in males (Monteiro-Fernandes et al., 2021). The cognitive performance also varies by sex and context, with both male and female advantages reported across studies, and stress-impairing memory in both sexes (Chaudry et al., 2022; Torrisi et al., 2023; Shirzadi et al., 2024).

## Neuronal Loss and Its Role in Cognitive Decline

Neuronal loss is a key feature of AD and correlates strongly with cognitive decline (Svenningsson et al., 2022). This loss exceeds neurofibrillary tangle accumulation and is not directly related to senile plaques (Zwang et al., 2024). Various mechanisms contribute to neuronal death, including apoptosis and caspase-6 activation (Theofilas et al., 2022). Neuronal loss is most severe in early AD stages and reaches a critical point in moderate AD before declining to a floor level in severe AD (Wegiel et al., 2021). Intraneuronal Aβ accumulation may play a role in neuronal loss in a transgenic mouse model (Wirths and Zampar, 2020). However, in mouse models without pathologic Tau, neuronal loss is restricted to the core of Aβ deposits and correlates with deposit size (Zhang et al., 2021). Understanding the dynamics of neuronal loss is crucial for identifying treatment targets and timing in AD (Wegiel et al., 2022).

## Strategic Drug Repurposing to Unlock New Treatments for Alzheimer’s Disease

Currently, no small-molecule therapeutics are available that effectively halt the progressive neurodegeneration characteristic of AD. A common strategy in drug discovery involves target-based screening, wherein disease-associated molecular targets, often derived from publicly available omics datasets, are systematically matched to drug databases to identify compounds with potential for therapeutic repurposing (Rodriguez et al., 2021).

In our studies, we employed a systems pharmacology framework that integrates structure-based, genome-scale off-target predictions with drug-induced gene expression profiles to screen both FDA-approved and investigational compounds for potential efficacy in AD (Hodes and Buckholtz, 2016; Lim et al., 2016). As a molecular signature of AD pathology, we used brain region-specific differential gene expression data comparing AD patients to cognitively healthy controls, obtained from the Accelerating Medicines Partnership for AD data portal (Hodes and Buckholtz, 2016). We also utilized the Library of Integrated Network-Based Cellular Signatures (LINCS; https://lincsproject.org/) consortium’s L1000 platform as a high-throughput gene expression profiling tool, which quantified mRNA levels of 978 landmark genes to capture transcriptional changes in cells in response to diverse perturbing agents.

To identify candidate compounds, the genome-wide AD gene expression signature was computationally matched to drug-induced transcriptional responses. Specifically, compounds that produced inverse expression patterns, upregulating genes downregulated in AD and downregulating genes upregulated in AD, were prioritized as candidates for therapeutic repurposing. This predictive methodology has been previously described (Lim et al., 2016; Meng et al., 2022).

## Rationale for Investigating Selected Treatments

Investigated treatments included six drugs: ibudilast, timapiprant, RG2833, combined diazoxide (DZ), dibenzoylmethane (DIB), and BT-11, each targeting distinct pathways.

### Ibudilast: Apotential neuroprotective agent

Ibudilast, a non-selective phosphodiesterase inhibitor, has demonstrated anti-inflammatory and neuroprotective properties. It suppresses pro-inflammatory cytokines, inhibits toll-like receptor 4 (TLR4), and promotes neurotrophic factors (Angelopoulou et al., 2022). A preclinical study shows that ibudilast enhances spatial learning, reduces AD pathology, and mitigates neuroinflammation in transgenic models (Oliveros et al., 2023). Ibudilast also exhibits protective effects against Aβ-induced neurotoxicity and memory impairment in mice (Wang et al., 2014). Although less effective in reducing focal inflammation in multiple sclerosis, it preserved brain volume and slowed disability progression (Goodman et al., 2016). Its ability to cross the blood–brain barrier, oral bioavailability, and safety profile highlights its potential for treating neurological conditions (Angelopoulou et al., 2022).

### Timapiprant: A selective PGD2 receptor antagonist

Neuroinflammation, marked by glial activation and elevated inflammatory mediators, is a hallmark of AD (Novoa et al., 2022). Prostaglandin D2 (PGD2) contributes to this process, and timapiprant, a selective PGD2 receptor (DP2) antagonist, has shown promise in reducing AD pathology and cognitive deficits (Wallace et al., 2022). Other pro-resolving lipid mediators, such as maresin 1 and resolvin D1, have exhibited neuroprotective effects and enhanced Aβ clearance (Miyazawa et al., 2020). Modulating pathways such as nuclear factor kappa B and mitogen-activated protein kinase signaling offer additional therapeutic avenues (Sivamaruthi et al., 2023). Polyphenolic compounds such as curcumin and resveratrol are also being explored for their anti-inflammatory and neuroprotective properties (Azzini et al., 2024; Moukham et al., 2024).

### RG2833: A histone deacetylase inhibitor

Histone deacetylase (HDAC) inhibitors are emerging as promising agents to mitigate cognitive decline in neurodegenerative disorders. RG2833, an HDAC1/3 inhibitor, improved spatial memory in female AD model rats and upregulated genes critical for synaptic plasticity (Ndukwe et al., 2025). Other HDAC inhibitors, such as vorinostat, sodium valproate, and sodium butyrate, have restored memory in AD models (Fernando et al., 2020). Inhibition of HDAC2, a key regulator of cognitive function, restored structural and synaptic plasticity (Burns and Graff, 2021; Pal et al., 2023). Selective inhibitors targeting class I HDACs demonstrate significant potential as cognitive enhancers (Burns and Graff, 2021).

### Diazoxide: Modulating cellular processes in Alzheimer’s disease

Diazoxide, traditionally used to treat hypoglycemia, modulates key cellular processes relevant to AD. It activates adenosine triphosphate-sensitive potassium channels, particularly in mitochondria, improving neuronal survival and cognitive function (Lv et al., 2022). Additionally, neuroprotective effects of diazoxide are mediated through p38 mitogen-activated protein kinase signaling, reducing endoplasmic reticulum stress and caspase-12 activation (Guan et al., 2018). It further prevents mitochondrial dysfunction by suppressing Bax translocation and inhibiting cytochrome c release, protecting neurons against ischemia-induced death (Lei et al., 2018). These findings highlight the therapeutic potential of diazoxide in treating AD and other neurodegenerative conditions.

### Dibenzoylmethane in Alzheimer’s disease

DIB has shown promise in mitigating spatial memory deficits, reducing Aβ plaques, and alleviating Tau pathology in AD models (Wallace et al., 2024). DIB demonstrates antioxidant effects, inhibits acetylcholinesterase (López-Iglesias et al., 2014), and interacts with early Aβ assemblies to prevent toxic oligomer formation (Frydman-Marom et al., 2009). A related compound, such as methylene blue, stabilizes mitochondria and exhibits neuroprotective properties (Atamna and Kumar, 2010). Other molecules, including 1,2-dihydroxybenzene-containing compounds and 2’,6’-dihydroxy-4’-methoxy dihydrochalcone, have demonstrated Tau aggregation inhibition and cognitive improvement in AD models (Soeda et al., 2015; Goncalves et al., 2021). While Wang et al. (2011) suggested cognitive improvements independent of Aβ-related mechanisms, further research is needed to elucidate the effects of DIB fully (**Table 1**).

**Table 1:** Summary of investigated treatments in TgF344-AD rats

Treatment	Mechanism of action	Reported effects in AD models
Ibudilast	PDE inhibitor; TLR4 suppression; neurotrophic support	Reduced Aβ pathology, neuroinflammation; improved memory
Timapiprant	DP2 (PGD2 receptor) antagonist	Reduced AD pathology; anti-inflammatory; restored cognition
RG2833	HDAC1/3 inhibitor	Enhanced synaptic genes; improved spatial memory
Diazoxide	KATP channel opener; mitochondrial protector	Reduced ER stress; improved neuronal survival and cognition
Dibenzoylmethane	Antioxidant; anti-Aβ aggregation; AChE inhibition	Reduced plaques, Tau; improved memory; antioxidant actions

AChE: Acetylcholinesterase; AD: Alzheimer’s disease; Aβ: amyloid-beta; DP2: prostaglandin D2 receptor 2; ER: endoplasmic reticulum; HDAC: histone deacetylase; PDE: phosphodiesterase; PGD2: prostaglandin D2; TLR: toll-like receptor.

## Exploring Novel Therapeutics

### BT-11: Applications beyond inflammatory diseases

BT-11, initially investigated for inflammatory bowel disease, targets the glutathione-s transferase lanthionine synthetase C-like 2 (LANCL2), to stabilize regulatory T cells via immunometabolic pathways (Leber et al., 2018). Leber et al. (2019) demonstrated its safety and tolerability, with Phase I trials confirming its minimal systemic exposure and promising anti-inflammatory effects. Although primarily researched in inflammatory diseases, the mechanisms of BT-11 may be relevant for AD treatment, warranting further investigation

BT-11, a small molecule targeting immunometabolic mechanisms, showed promising results in improving cognitive performance in male TgF344-AD rats (Birnbaum et al., 2024). Specifically, male rats treated with BT-11 exhibited enhanced spatial learning abilities and increased neuronal density, suggesting that these treatments may help preserve synaptic function and reduce neuronal loss (Birnbaum et al., 2024; Wallace et al., 2024). These findings are significant because they demonstrate that targeting synaptic plasticity and neurotransmission can help preserve neuronal integrity in AD models. However, these benefits were not observed in female rats, who did not show significant cognitive deficits or neuronal loss (Birnbaum et al., 2024). This sex-dependent response highlights the need for sex-specific treatment approaches, as treatments that work in males may not have the same effect in females.

### Ibudilast

Ibudilast, a drug with anti-inflammatory properties, was assessed for its potential to reduce neuroinflammation and improve cognitive function in TgF344-AD rats. The results were mixed, with ibudilast showing some improvements in spatial learning during the early acquisition phases of cognitive tasks (Oliveros et al., 2023). These cognitive benefits were accompanied by reductions in Aβ plaque load, but not on neuronal loss. Interestingly, ibudilast treatment led to a decrease in the ratio of amoeboid to ramified microglia in the hippocampal dentate gyrus (DG), suggesting that it may have some anti-inflammatory effects in the brain. The lack of significant effects on neuronal survival, however, suggests that benefits of ibudilast may be limited to specific stages of cognitive decline and may not, by itself, fully address the underlying pathological changes in AD (Oliveros et al., 2023).

### RG2833

RG2833, a compound that inhibits HDAC activity, showed genotype- and sex-dependent effects, with significant improvements in cognitive performance, only observed in female TgF344-AD rats (Ndukwe et al., 2025). These cognitive benefits occurred without a corresponding reduction in Aβ plaque load or microgliosis, suggesting that RG2833 may exert its effects through mechanisms other than Aβ plaque clearance or inflammation modulation (Ndukwe et al., 2025). In females, the ability of RG2833 to enhance cognitive function without altering pathological markers suggests that it may act through processes such as synaptic plasticity or neurogenesis, which could offer novel therapeutic avenues for AD.

## Timapiprant Treatment and Its Impact on Neuronal Loss

The results of the timapiprant treatment in TgF344-AD rats provide valuable insights into the potential therapeutic effects of DP1 receptor antagonists in AD. In TgF344-AD rats, DP1 receptor level was elevated in microglia, especially in the DG-hilar region, where microgliosis was also more pronounced (Wallace et al., 2022). Notably, timapiprant treatment led to a significant improvement in the active place avoidance task, suggesting that DP1 modulation can ameliorate cognitive deficits in TgF344-AD rats.

Additionally, timapiprant treatment reduced key pathological features in the hippocampus, including Aβ plaque burden, neuronal loss, and microgliosis in the DG-hilar region. This suggests that the action of timapiprant on microglial activation and neuroinflammation could play a critical role in reducing neurodegeneration in AD. Moreover, treatment led to a reduction in DP1 receptor levels in the DG-hilar region, which may contribute to the observed improvements in cognitive function and neuropathology. These results underscore the potential of targeting neuroinflammation to reduce neuronal loss and slow disease progression in AD.

## Effects of Other Treatments on Neuronal Loss and Tau

In terms of Tau-related pathology, several treatments showed the potential to modulate p-Tau levels. BT-11 and DZ/DIB were evaluated for their ability to affect p-Tau in addition to their effects on neuronal loss. While both treatments improved cognitive performance, BT-11 reduced neuronal loss, but had no impact on p-Tau in male TgF344-AD rats (Birnbaum et al., 2024). DZ/DIB reduced p-Tau but did not affect neuronal loss (Birnbaum et al., 2024; Wallace et al., 2024). This suggests that while these compounds may help mitigate cognitive deficits, their effects on Tau pathology and neuronal survival may be limited or may require more specific targeting of Tau-related pathways.

In addition, RG2833, which inhibits HDAC activity, showed no genotype- and sex-dependent effects on neuronal loss. Female TgF344-AD rats treated with RG2833 exhibited some improvement in cognitive function, though no significant reduction in neuronal loss was observed (Ndukwe et al., 2025). The lack of changes in neuronal loss suggests that effects of RG2833 on cognitive function may not be linked to neuronal loss but could be mediated by other mechanisms, such as synaptic plasticity or neurogenesis, which may indirectly contribute to reducing neuronal loss (**Table 2**).

**Table 2:** Summary of therapeutic interventions in TgF344-AD rats

Treatment	Observed effects	Mechanistic insight / notes
BT-11	Improved cognition and neuronal density (males only); no effect on Tau	LANCL2 modulator; immunometabolic pathway
Ibudilast	Reduced amyloid load; mild cognitive benefit; no neuronal rescue	PDE inhibitor; anti-inflammatory; lowered microgliosis
RG2833	Cognitive benefit in females only; no effect on Aβ, microgliosis, or neuronal loss	HDAC1/3 inhibition; possible action via synaptic plasticity
Timapiprant	Improved cognition; reduced Aβ, neuronal loss, and microgliosis	DP1 receptor antagonist; lowered microglial activation in DG-HL
DZ/DIB	Reduced Tau hyperphosphorylation; no effect on neuronal loss	Tau-targeting potential; cognitive improvement observed

AD: Alzheimer’s disease; Aβ: amyloid-beta; DG-HL: dentate gyrus-hilar; DIB: dibenzoylmethane; DP1: prostaglandin D2 receptor 1; DZ: diazoxide; HDAC: histone deacetylase; LANCL2: lanthionine synthetase C-like 2; PDE: phosphodiesterase.

## Implications for Sex-Specific Therapeutic Strategies

Our findings highlight the importance of considering sex as a biological variable in AD research and treatment development. The studies suggest that sex differences in cognitive performance, neuropathology, and treatment response are critical factors that may need to be incorporated into the design of clinical trials and therapeutic interventions. For example, treatments that are effective in male AD models may not produce the same results in females, and *vice versa*. This underscores the need for more targeted, personalized approaches to treating AD, where sex-specific factors are considered.

Moreover, the differential effects of treatments on microgliosis, neuronal loss, synaptic plasticity, Tau pathology, and neuroinflammation suggest that modulating neuroinflammation, preserving synaptic function, and reducing neuronal loss could be key strategies in addressing cognitive decline in AD. Future research should focus on understanding the underlying molecular mechanisms that drive sex differences in AD pathology, as well as identifying biomarkers that can predict treatment response in different sexes.

## Sex Differences in Neuronal Loss, Microglial Function, and Hormones on Alzheimer’s Disease Pathology

Research indicates significant sex differences in AD, with women showing higher risk and potentially greater cognitive decline (Santiago and Potashkin, 2023). These differences may be attributed to various factors, including sex-specific neuronal loss patterns (Poon et al., 2023), microglial function (Delage et al., 2021), and hormonal influences (Rahman et al., 2019). Estrogen loss in postmenopausal women is a potential contributor to AD risk, affecting β-amyloid precursor protein (APP) processing and brain-derived neurotrophic factor regulation (Bagit et al., 2021). Neuronal loss correlates with cognitive impairment and exceeds neurofibrillary tangle accumulation in AD (Zwang et al., 2024). Interestingly, women exhibit more AD pathology than men, particularly neurofibrillary tangles, and show a stronger correlation between pathology and clinical AD diagnosis (Santiago and Potashkin, 2023). Animal studies support these findings, demonstrating sex-related differences in amyloid deposition and cognitive impairment onset (Poon et al., 2023).

Microglia play a crucial role in the sexual dimorphism observed in AD, with women having a higher risk and potentially experiencing greater cognitive decline (Delage et al., 2021). Sex differences in microglial function, metabolism, and gene expression contribute to this disparity (Guillot-Sestier et al., 2021; Kadlecova et al., 2023). Female microglia exhibit increased glycolytic activity, reduced phagocytosis, and higher expression of inflammatory genes, potentially exacerbating AD pathology (Guillot-Sestier et al., 2021; Coales et al., 2022; O’Neill et al., 2022). These differences may be influenced by sex hormones, aging, and genetic factors such as APOE genotype (Chen et al., 2021). Microglial sex differences persist throughout the lifespan and affect various neurodevelopmental processes (Kodama and Gan, 2019). Understanding these sex-specific microglial characteristics could provide insights into AD pathogenesis and potential therapeutic targets for sex-specific interventions in neurodegenerative diseases.

Estrogen plays a significant role in AD pathology in rodent models. Farkas et al. (2022) showed that estrogen deficiency accelerates Aβ plaque formation and increases Aβ production. Estrogen replacement therapy has demonstrated neuroprotective effects, including enhanced neurotrophin signaling, synaptic activity, and protection against Aβ toxicity (Guo et al., 2020). Estrogen decreases Aβ generation and secretion by increasing intracellular trafficking of APP (Xu et al., 2006). It also promotes non-amyloidogenic processing of APP and reduces plaque burden (Amtul et al., 2010). The estrogen receptor β is particularly important in regulating neurological health and has been implicated in AD development (Zhao et al., 2015). However, the timing of estrogen therapy is crucial, as late intervention may increase dementia risk (Merlo et al., 2017). These findings highlight the complex role of estrogen in AD pathology and the potential for targeted interventions in rodent models.

## Impact of Drug Treatments on the Transcriptome of TgF344-Alzheimer’s Disease Rats

**Figure 2:**
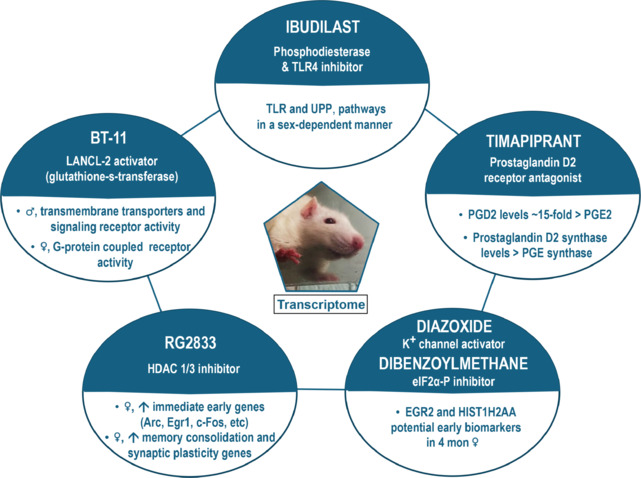
Schematic summary of transcriptomic effects of five pharmacological treatments in TgF344-AD rats. The figure illustrates the impact of ibudilast, timapiprant, diazoxide/dibenzoylmethane (combination therapy), RG2833, and BT-11 on transcriptomic alterations relevant to AD in the TgF344-AD rat model. Each drug is represented within a distinct circle arranged in a pentagonal configuration. Pharmacological targets are indicated in the dark blue segment at the top of each circle. Where observed, sex-specific responses to treatment are annotated. At the center, a representative image of a TgF344-AD rat is shown alongside the AD-relevant phenotype assessed: transcriptomic alterations. Drug-induced changes in gene expression were determined relative to untreated TgF344-AD controls. The white portion of each circle summarizes affected molecular pathways. Detailed descriptions of the effects of each compound are available in the referenced publications. AD: Alzheimer’s disease; Arc: activity-regulated cytoskeletal-associated protein; Egr1: early growth response 1; EGR2: early growth response 2; eIF2α-P: phosphorylated eukaryotic initiation factor 2; HDAC: histone deacetylase; HIST1H2AA: histone H2AA; LANCL-2: lanthionine synthetase C-like 2; PGD2: prostaglandin D2; PGE(2): prostaglandin E(2); TLR: toll-like receptor; UPP: ubiquitin proteasome pathway.

In this section, we discuss the effects of the five treatments on gene expression and their canonical pathways using the TgF344-AD rat model of AD (**Figure 2**). RNA sequencing (RNAseq) analyses were conducted to evaluate gene expression in drug-treated compared to untreated TgF344-AD rats. The whole left hippocampal tissue was prepared as described in (Wallace et al., 2022) and used for RNA sequencing analysis, which was outsourced to the UCLA Technology Center for Genomics & Bioinformatics (Los Angeles, CA, USA). Gene expression was compared between samples from five drug-treated to five untreated transgenic rats (both male and female). The gene expression data were normalized to reads per million using the Trimmed Mean of M-values method. Differentially expressed genes between drug-treated and untreated TgF344-AD rats for each sex were identified using the edgeR program. Reads per millions were analyzed for fold-change, P-values, and false discovery rate for each gene. Further analysis and visualization were carried out using R Studio (Love et al., 2014; Team, 2023), accessed December 6, 2023.

### Ibudilast

Ibudilast is known to target multiple pathways, and the RNAseq results demonstrated that ibudilast treatment reduced inflammation by lowering the expression of several pro-inflammatory proteins, including cytokine interleukin (IL)-6, interferon receptor 1, macrophage migration inhibitory factor, microglial proteins such as transmembrane protein 119, arachidonate 5-lipoxygenase activating protein, toll-like receptor 7, lysosomal protein transmembrane 5, and triggering receptor expressed on myeloid cells 2. Additionally, a TLR4 antagonist further supported this reduction in pro-inflammatory factors (Oliveros et al., 2023).

Further analysis showed that ibudilast treatment led to increased expression of synaptic proteins such as synaptotagmin 2, syntaxins, and cadherin-1, all of which participate in synaptic structure and function. Interestingly, ibudilast also decreased the expression of brain-derived neurotrophic factor two-fold in both male and female-treated rats, suggesting that ibudilast provides neuroprotection through mechanisms independent of growth factor upregulation.

These analyses also generated gender-specific differences. In males, the most affected canonical pathways included axonal guidance, G-protein coupled receptor signaling, synaptogenesis signaling, cyclic adenosine monophosphate-mediated signaling, and neuroinflammation signaling. In females, ibudilast treatment influenced pathways related to synaptogenesis, white adipose tissue browning, calcium signaling, corticotropin-releasing hormone signaling, and gonadotropin-releasing hormone signaling.

A deeper RNAseq analysis revealed gender-specific differences in the expression of TLR and ubiquitin-proteasome pathways following ibudilast treatment, a novel finding not previously reported in AD models. These results suggest that ibudilast could serve as a repurposed drug to target multiple pathways, including TLR signaling and the ubiquitin/proteasome pathway, to mitigate cognitive deficits and AD pathology. For example, ibudilast-induced upregulation of interleukin-1 receptor-associated kinase 3 could prevent nuclear factor kappa B activation, with components of the TLR pathway dependent on degradation by the ubiquitin/proteasome pathway for nuclear factor kappa B nuclear translocation and activation.

Other studies have examined the effects of ibudilast on neurodegeneration and reported comparable alterations in gene expression profiles (reviewed in Angelopoulou et al., 2022; Chen et al., 2024). Notably, ibudilast has been shown to promote autophagy and lysosomal biogenesis via activation of the mammalian target of rapamycin complex 1–transcription factor EB signaling axis in neuronal-like cell models (Chen et al., 2020). At the mechanistic level, known molecular targets of ibudilast, such as phosphodiesterases PDE3A, PDE4B, and PDE4D, have been reported to physically interact with proteins encoded by several putative AD risk genes, including *BIN1*, *FYN*, and *GSK3*β (Xu et al., 2022).

### Timapiprant

Timapiprant, a selective DP1 receptor antagonist, was studied to assess its impact on PGD signaling in the context of AD (Wallace et al., 2022). PGD2, the most abundant prostaglandin in the brain, is known to increase under neuropathological conditions. While the literature on the relevance of PGD2 to AD is limited, studies have indicated that the PGD2 pathway could play a role in modulating AD-related pathology.

Using the TgF344-AD transgenic rat model, which exhibits age-dependent and progressive AD pathology, including neuronal loss and gliosis, RNAseq analyses revealed that PGD2 levels were significantly higher than prostaglandin E2 in the hippocampus of both TgF344-AD and wild-type rats. The primary PGD2 synthase, lipocalin-type PGDS, was found to be the most highly expressed among 33 genes involved in the PGD2 and PGD2 pathways. Treatment with timapiprant, significantly mitigated AD pathology and cognitive deficits in TgF344-AD males.

Interestingly, PGD2 signals through DP1 and DP2 receptors, and in TgF344-AD rats, microglia displayed enhanced DP1 receptor expression, while neuronal DP2 receptors were fewer compared to wild-type rats. This suggests a role for PGD2 signaling in modulating microglial activation and neuroinflammation in AD, potentially providing an alternative therapeutic target to reduce plaque load, neuronal loss, and microgliosis in AD patients.

In a separate study, administration of the prostaglandin E2 receptor 2 antagonist TG11-77.HCl to female 5xFAD mice significantly reduced the mRNA expression of proinflammatory cytokines (IL-1β, tumor necrosis factor, IL-6, C-C motif ligand 2, and prostaglandin E2 receptor 2) and glial activation markers (Iba1, glial fibrillary acidic protein, CD11b, and S100B) that were upregulated following lipopolysaccharide stimulation (Banik et al., 2021). Emerging evidence from recent investigations in amyotrophic lateral sclerosis further supports the role of prostaglandin receptor-mediated signaling pathways in promoting neurotoxicity within the central nervous system (reviewed in Nango et al., 2023). Elucidating the contribution of prostaglandin signaling to neurodegenerative processes may advance our understanding of disease pathophysiology and inform the development of targeted therapeutic strategies for disorders such as AD.

### Diazoxide/dibenzoilmethane

Around two-thirds of AD patients are women, highlighting the importance of identifying early biomarkers that can signal the initial stages of AD, when interventions are most effective. To investigate this, we first examined gene expression in untreated TgF344-AD and wild-type female rats at 4 months of age, a pre- or early pathology stage of AD. RNAseq analysis revealed significant differences in gene expression between TgF344-AD and wild-type females, including the upregulation of *APPsw* and *PS1-∆E9*, hallmark genes of the TgF344-AD model. Additionally, we identified two potential early biomarker genes for AD, early growth response 2 and histone H2AA, in the hippocampus of 4-month-old TgF344-AD females. Among the 17,168 genes analyzed, only these two genes showed significant expression changes compared to their wild-type littermates, besides *APPsw* and *PS1-∆E9* (Wallace et al., 2024).

We also compared gene expression between DZ/DIB-treated and untreated TgF344-AD females at 4 months of age. Interestingly, DZ/DIB treatment led to the upregulation of genes involved in neurogenesis, differentiation, synaptic plasticity, apoptosis, and amyloid toxicity. Genes such as *OLFM3* (olfactomedin 3, involved in brain and retina differentiation), *SLC17A6* (vesicular glutamate transporter 2, important for synaptic plasticity), *CNTN6* (contactin 6, associated with axonal formation), and *IRF6* (interferon regulatory factor 6, relevant to apoptosis regulation) were upregulated in treated rats. This suggests that DZ/DIB treatment could mitigate early-stage AD pathology and prevent cognitive decline by upregulating genes known to be downregulated in AD and aging.

To date, no published studies have investigated the combined effects of DZ and DIB in the context of neurodegenerative diseases, including AD. However, monotherapy with DZ has been shown to attenuate microgliosis when administered either before or after motor neuron injury in murine models (Nogradi et al., 2020). Similarly, DIB treatment alone has been reported to suppress adiposity-induced inflammatory and oxidative responses, as well as inflammation-mediated neuronal cell death (You and Choi, 2023), and to exert protective effects in mouse models of stress and neurodegeneration (Sambon et al., 2020). Recent investigations into the mechanisms of action of DIB and structurally related compounds have highlighted their potential as novel therapeutic agents for neurodegenerative disorders (reviewed in Bettendorff, 2023). Despite these findings, there remains a paucity of data regarding the therapeutic efficacy of DZ and DIB, either individually or in combination, in neurodegenerative disease models. Further studies are warranted to elucidate their mechanisms and evaluate their therapeutic potential, particularly in the context of AD.

### RG2833

The HDAC 1/3 inhibitor RG2833 was evaluated for its potential to improve hippocampal-dependent spatial memory in 11-month TgF344-AD females. Gene expression analysis via RNAseq revealed 358 differentially expressed genes in females, with no such changes in males. Specifically in females, RG2833 treatment upregulated immediate early genes such as *Arc*, *Egr1*, and *c-Fos*, and other genes that participate in memory consolidation and synaptic plasticity (Ndukwe et al., 2025).

Pathway analysis showed that upregulated genes were associated with memory, estrogen regulation, and AD, with notable gene changes linked to HDAC inhibition and memory improvement. RG2833 downregulated genes involved in drug efflux transport and metabolism, which could lead to intracellular accumulation of the drug and enhanced efficacy.

Interestingly, when applying the same criteria for gene expression analysis as used for females, no significant changes were observed in males, suggesting a potential sex-specific mechanism of action. These findings suggest that RG2833 may be particularly effective in improving cognitive function in female AD patients.

RG2833 was considered as a therapy for Friedreich ataxia, a rare and the most common inherited ataxia. Friedreich ataxia results from transcriptional silencing of the nuclear *FXN* gene, which encodes the mitochondrial protein frataxin (Gottesfeld, 2019). A 2013 Phase I trial of the HDAC inhibitor RG2833 showed proof-of-concept but was discontinued due to pharmacokinetic and safety issues. It should be noted that in our long-term (6 months) chronic studies with the TgF344-AD rats no toxicity was observed. The follow-up compound of BioMarin, BMN 290, exhibited improved brain penetration and stability in preclinical models but was also discontinued in 2019 (Gottesfeld, 2019). Alternative therapeutic approaches are under investigation.

### BT-11

BT-11, an investigational drug that reduces gut inflammation and improves cognitive function, was evaluated for its effects on gene expression and pathway enrichment in BT-11 treated compared to untreated TgF344-AD rats (Birnbaum et al., 2024). The treatment led to significant changes in gene expression, particularly in males (195 genes) and females (77 genes). Pathway enrichment analyses revealed that males exhibited enriched pathways related to transmembrane transporters and signaling receptor activity, while females showed enriched pathways related to G-protein coupled receptor activity.

BT-11 treatment also induced changes in the cyclic adenosine monophosphate pathway, suggesting its role in modulating hippocampal signaling. Notably, LANCL2, a target of BT-11, was localized in oligodendrocytes in the hippocampus but not in neurons, astrocytes, or microglia. These findings suggest that BT-11 has a promising potential to treat AD by modulating neuroinflammation and supporting hippocampal function, particularly in males with reduced Aβ plaque load and neuronal loss.

A Phase I clinical trial evaluated the safety, tolerability, and pharmacokinetic profile of orally administered BT-11 in healthy adult volunteers. The compound was well-tolerated across all dosing cohorts, with no serious adverse events observed (Leber et al., 2020). Preliminary findings support the potential of BT-11 as a cognitive-enhancing and neuroprotective agent, particularly in the context of AD. Its mechanism of action involves modulation of the LANCL2 signaling pathway, which is associated with attenuation of neuroinflammatory responses. These findings warrant further investigation through additional preclinical and clinical studies to better characterize the compound’s mechanisms of action and therapeutic efficacy in neurodegenerative disorders (**Table 3**).

**Table 3:** Transcriptomic effects of AD treatments

Treatment	Transcriptomic effects	Notes
Ibudilast	Downregulated pro-inflammatory genes (e.g., *IL-6, MIF, TLRs*); Upregulated synaptic genes (*SYT2, STX, CDH1*); sex-specific pathways (e.g., neuroinflammation, synaptogenesis); altered TLR/UP signaling	Targets PDEs, TLR signaling, and ubiquitin-proteasome pathway; promotes synaptic function and lysosomal biogenesis
Timapiprant	Modulated PGD2 signaling; reduced microglial DP1 expression; supported reduction of neuroinflammation markers; enriched PGDS expression in hippocampus	DP1 receptor antagonist; alters glial inflammatory pathways; potential sex-specific signaling patterns
DZ/DIB	Upregulated genes for neurogenesis and synaptic plasticity (e.g., *OLFM3, SLC17A6*); identified early biomarker candidates (*EGR2, HIST1H2AA*); potential for early intervention	No prior studies on combination therapy in AD; indicates neuroprotective and gene regulatory potential in females
RG2833	358 DEGs in females only; upregulated memory-related immediate early genes (*Arc, Egr1, c-Fos*); pathways related to estrogen, memory, and HDAC inhibition	Effective in females; no toxicity in chronic studies; pharmacokinetic issues in human trials noted
BT-11	195 DEGs in males, 77 in females; enriched GPCR and transporter pathways; LANCL2 expression localized to oligodendrocytes; modulated cAMP signaling	Modulates LANCL2; safe in Phase I trial; strong male-specific effects in AD model

AD: Alzheimer’s disease; Arc: activity-regulated cytoskeletal-associated protein; cAMP: cyclic adenosine monophosphate; DEGs: differentially expressed genes; DIB: dibenzoylmethane; DP1: prostaglandin D2 receptor 1; DZ: diazoxide; Egr1: early growth response 1; GPCR: G protein-coupled receptor; HDAC: histone deacetylase; IL6: interleukin-6; LANCL2: lanthionine synthetase C-like 2; MIF: macrophage migration inhibitory factor; PDE: phosphodiesterase; PGD2: prostaglandin D2; PGDS: prostaglandin D synthase; STX: syntaxins; SYT2: synaptotagmin 2; TLR: toll-like receptor; UP: ubiquitin proteasome.

## Conclusions

Our work explored the effects of five distinct treatments — ibudilast, timapiprant, RG2833, DZ, and DIB combined, and BT-11 — on cognitive deficits, neuronal loss, neuropathology, and gene expression in TgF344-AD rats. Each treatment was selected based on its ability to target key pathological mechanisms associated with AD, including neuroinflammation, synaptic dysfunction, and histone deacetylation. Our findings emphasize the importance of sex differences in AD pathology and treatment efficacy.

Our work highlights two key innovations: examining sex-specific differences in AD and using drug repurposing to accelerate treatment discovery. By stratifying preclinical data by sex and integrating machine learning with *in vivo* validation, we enhance translational relevance and advance precision medicine in AD research.

Moreover, the differential effects of these treatments highlight the multifaceted nature of AD pathology and the need for targeted therapeutic interventions. While some treatments effectively reduced neuroinflammation and improved cognitive function, their impact on amyloid plaques, Tau pathology, and neuronal survival varied. It suggests that effective AD treatment may need combination therapy. The observed sex-dependent responses further emphasize the need for personalized medicine approaches in AD treatment. Future research should focus on optimizing these therapeutic strategies and exploring their translational potential in clinical settings.

**Additional file:** *Open peer review report 1.*

OPEN PEER REVIEW REPORT 1

## Data Availability

*Not applicable*.
