# Supplementary material for: Six promising drug repurposing candidates for Alzheimer’s disease and their sex-specific mechanisms and efficacy
Source: Neural Regen Res. 2025 Aug 13;21(7):2882–8. doi: 10.4103/NRR.NRR-D-25-00256 (PMC13378927; doi:10.4103/NRR.NRR-D-25-00256)
Supplement: Supplementary file 1 [file NRR-21-2882_Suppl1.pdf]

OPEN PEER REVIEW REPORT 1

Name of journal: Neural Regeneration Research
Manuscript NO: NRR-D-25-00256
Title: Drug Repurposing for Alzheimer's disease: Six Promising Candidates in Focus
Reviewer’s Name: Takayoshi Ubuka
Reviewer’s country: Japan

COMMENTS TO AUTHORS

This manuscript reviewed some novel drug candidates for Alzheimer's disease (AD) summarizing their effects on the TgF344-AD rat model. The drugs investigated were ibudilast, timapiprant, RG2833, diazoxide/dibenzoylmethane (combined), and BT-11. It was not clear from this manuscript why these 5 treatments were selected. The authors state that computational methods and large-scale biological datasets were utilized but it is important to explain in a logical manner about why these drugs were selected and why others were not selected.

Explaining the sex differences in AD pathology, neurophysiology and the effects of the treatments were interesting and should be useful in future study and to develop the treatment of AD.

It is novel but the rationale should be explained.
